# Draft genome sequence of *Gordonia lacunae* iso11, a polypropylene-degrading bacterium isolated from a peat bog

**DOI:** 10.1128/mra.00802-25

**Published:** 2025-09-08

**Authors:** Scott A. Coughlin, Stephen A. Kelly, Julianne Megaw

**Affiliations:** 1 School of Biological Sciences, Queen’s University Belfast, Belfast, United Kingdom; 2 School of Pharmacy, Queen’s University Belfast, Belfast, United Kingdom; 3 School of Medicine and Centre for Interventions in Infection, Inflammation, and Immunity (4i), University of Limerick 8808, Limerick, Ireland; Rochester Institute of Technology, Rochester, New York, USA

**Keywords:** *Gordonia*, plastic biodegradation, polypropylene, bioremediation

## Abstract

We report the draft genome sequence of *Gordonia lacunae* iso11, a polypropylene-degrading bacterium isolated from a peat bog in Northern Ireland. Sequencing identified a 5,845,760 bp genome with 67.98% GC content, and annotation revealed several genes associated with hydrocarbon and plastic biodegradation, highlighting its potential for their bioremediation.

## ANNOUNCEMENT

Members of the bacterial genus *Gordonia* are found in diverse terrestrial, aquatic, and clinical environments (1). *Gordonia* spp. are known for their metabolic versatility, particularly relating to their ability to degrade a wide variety of carbon sources (2) including hydrocarbons (1–4) and plastics such as polystyrene, polyethylene, and polyethylene terephthalate (5–8), making them valuable to bioremediation efforts.

*Gordonia* sp. iso11 was isolated from a peat bog in Northern Ireland (54.4878774° N, 6.6129615° W). A 5 g peat sample was suspended in 25 mL 5% wt/vol NaCl in a 50 mL centrifuge tube, and after sedimentation, 1 mL of the supernatant was used to inoculate 100 mL Erlenmeyer flasks containing 25 mL carbon-free Czapek-Dox medium composed of, in g L^−1^: NaNO_3_ (3.0), MgSO_4_ (0.5), KCl (0.5), K_2_PO_4_ (1.0), and FeSO_4_ (0.01), supplemented with 2.5 g polypropylene pellets as the sole carbon source. Flasks were incubated aerobically at 30°C and 100 rpm for 28 days, and growth was determined by observing turbidity of the otherwise clear and colorless medium. Pellets were removed and used to inoculate Czapek-Dox agar containing 0.5% wt/vol D-glucose. Plates were incubated at 30°C, and any resulting colonies were isolated by re-streaking onto plates of the same medium until pure cultures were obtained. Based on 16S rRNA gene sequencing, iso11 was confirmed as a member of the genus *Gordonia*.

Iso11 was observed to effectively degrade polypropylene (9), necessitating genome sequencing to provide mechanistic insights. For DNA extraction, iso11 was cultured in M9 minimal salts medium containing 0.5% wt/vol D-glucose for 48 h. DNA was extracted using the Merck GenElute Bacterial Genomic DNA Extraction Kit, using the protocol for Gram-positive bacteria.

Sequencing was performed by MicrobesNG (Birmingham, UK). Genomic DNA libraries were prepared using the Nextera XT Library Prep Kit (Illumina, San Diego, USA) following the manufacturer’s protocol with the following modifications: input DNA was increased twofold, and PCR elongation time was increased to 45 s. Libraries were sequenced on an Illumina Novaseq 6000, using a 250 bp paired-end protocol.

All tools for assembly and analysis were run using default parameters unless otherwise specified. Reads were quality- and adapter-trimmed using Trimmomatic v0.30 (10) with a sliding window cutoff of Q15. Reads were assembled *de novo* using SPAdes v3.7 (11), and contigs < 1000 bp were discarded. The genome was then annotated using the National Center for Biotechnology Information Prokaryotic Genome Annotation Pipeline v6.9 (12), which also identified the species as *G. lacunae*.

Sequencing yielded 2,266,224 reads with 183 × coverage. The assembled genome was 5,845,760 bp in 41 contigs, with an N50 of 335,840, L50 of 5, GC content of 67.98%, and 100% completeness, as determined by CheckM v1.2.3 (13).

Genome annotation identified multiple genes coding for proteins capable of cleaving or oxidizing C-C and C-H bonds, notably alkane-1-monooxygenase (AlkB), which is associated with biodegradation of both alkanes and plastics (14), highlighting the broad potential of this strain for bioremediation.

## Data Availability

The raw Illumina reads for iso11 have been deposited in the Sequence Read Archive (SRA) under the accession number SRR32034605. This Whole Genome Shotgun project has been deposited at DDBJ/ENA/GenBank under the accession JBLKRZ000000000. The version described in this paper is version JBLKRZ010000000.
